# The Flight Apparatus of Migratory and Sedentary Individuals of a Partially Migratory Songbird Species

**DOI:** 10.1371/journal.pone.0051920

**Published:** 2012-12-19

**Authors:** Adam M. Fudickar, Jesko Partecke

**Affiliations:** 1 Max Planck Institute for Ornithology, Radolfzell, Germany; 2 Department of Biology, University of Konstanz, Konstanz, Germany; Liverpool John Moores University, United Kingdom

## Abstract

Variations in the geometry of the external flight apparatus of birds are beneficial for different behaviors. Long-distance flight is less costly with more pointed wings and shorter tails; however these traits decrease maneuverability at low speeds. Selection has led to interspecific differences in these and other flight apparatuses in relation to migration distance. If these principles are general, how are the external flight apparatus within a partially migratory bird species shaped in which individuals either migrate or stay at their breeding grounds? We resolved this question by comparing the wing pointedness and tail length (relative to wing length) of migrant and resident European blackbirds (*Turdus merula*) breeding in the same population. We predicted that migrant blackbirds would have more pointed wings and shorter tails than residents. Contrary to our predictions, there were no differences between migrants and residents in either measure. Our results indicate that morphological differences between migrants and residents in this partially migratory population may be constrained.

## Introduction

Natural selection leading to fixed seasonal migration in animals has resulted in a multitude of behavioral and morphological adaptations that aid animals in the energetic demands of sustained movement over extended periods [1]. Birds provide an exceptional example of adaptation to migration. Selection has led to increases in the aerodynamic efficiency of external morphology, modified respiratory capacity, and seasonal plasticity in muscle and gut morphology of migratory birds [2]–[7]. Additionally, behavioral traits of migratory birds, such as flock formation, can increase individual flight efficiency [8].

Adaptations of the external flight apparatus for migration have been documented extensively in both interspecies and population level comparisons [2]–[3], [9]–[11]. Long distance migratory species have external morphological traits that increase the efficiency of forward flight whereas nonmigratory species have traits associated with maneuverability at low speeds [2]. As migration serves as a major bottleneck in the annual survival of migratory birds [12], it stands to reason that any trait that increases the potential for surviving migration could be strongly selected for. Population comparisons of external flight apparatuses have shown that traits which increase the efficiency of long distance flight correlate with increases in migration distance [3], [9]. Sympatrically breeding Central European blackcaps (*Sylvia atricapilla*) in southwestern Germany have undergone phenotypic divergence in less than 30 generations in response to divergence in migratory behavior [13]–[14]. Blackcaps breeding at the divide, which migrate in two general directions, have diverging wing morphology. Blackcaps that migrate ∼1090 km to Britain have rounder wings than their sympatrically breeding counterparts that migrate ∼1645 km to Spain [14]. Phenotypic variation in wing shape of sympatrically breeding blackcaps is remarkable given the relatively small change in migration distance and short time since divergence.

In the current study we compare the external flight apparatus of migrant and sedentary European blackbirds (*Turdus merula*) breeding within the same population (partially migratory population). Partial migration, when a breeding population consists of both migrant and resident individuals, is considered an intermediate stage between fixed migratory and fixed sedentary behavior at the population and species level [1], [15]–[16]. Therefore, partial migration provides the opportunity to gain insight into selective pressures leading to fixed migratory and sedentary phenotypes. Using a combination of radio telemetry and light level geolocation we tracked free living partially migratory blackbirds year round. By tracking individuals year round, we were able to accurately classify individuals as migrant or resident.

Blackbirds from the study area in southwestern Germany migrate on average 800 km west-southwest however, birds banded in the region during the breeding season have been recovered up to 2000 km away from the breeding site during the non breeding season [17]. Partecke and Gwinner [18] found that blackbirds breeding in two populations in Germany with known differences in the number of migrants in the wild, showed different levels of “migratory disposition” when reared under similar conditions in captivity. Combined, distances traveled by migratory blackbirds and the apparent endogenous control of migratory disposition suggest that differences in migration strategies in blackbirds in the region could be the result of genetic differences. However, as Partecke and Gwinner [18] found, female blackbirds in our study population are more likely to migrate than males (Fudickar and Partecke, unpublished data). Furthermore, there are no differences in the age makeup of migratory and sedentary individuals, male or female (Fudickar and Partecke, unpublished data). Therefore, migration in blackbirds seems to be a female-biased intrinsic trait.

Given the relatively long distance that migrant blackbirds from the region move seasonally compared to their non migratory counterparts, we predicted that migrant blackbirds would have their flight apparatus more adapted to long distance flight than year round residents in the same population. We compared two primary indices of the external flight apparatus of migrants and residents: wing pointedness and tail-wing ratio. Wing pointedness is frequently used as a measure of flight efficiency in birds. As wings become more pointed, long distance flight becomes more efficient [4]. In addition to pointed wings, migratory passerines typically have shorter tails (relative to wing length) than non migrants. Although longer tails aid in maneuvering through cluttered habitat, shorter tails reduce drag and increase the efficiency of forward flight [19]. Therefore, we also predicted that migrants would have smaller tail-wing ratio scores than residents. Further, if there is selection on migrants for more pointed wings and shorter tails, we predicted that wing pointedness and tail-wing ratio would be correlated.

## Results

Over three breeding seasons, we were able to assign wing pointedness scores to 131 blackbirds that were successfully tracked through the autumn. Out of 131 blackbirds, 82 were male and 49 were female (Table 1). Sixty individuals were classified as juveniles and 56 were classified as adults in the breeding season in which their feathers were measured. Out of 131 blackbirds that were assigned wing pointedness scores, 38 migrated during the autumn (29% migrants). Out of 75 individuals that we attained a tail-wing ratio score for, 50 were male and 25 were female. Thirty two individuals were classified as juveniles and 38 were classified as adults in the breeding season in which their feathers were measured. Of the 75 individuals that we attained a tail-wing ratio score for, 24 were autumn migrants (32%). Twenty migrants were observed on the breeding grounds the spring following migration of which 17 were recaptured. Three returned migrants were observed at the breeding grounds but were never successfully recaptured. Two recaptured birds lost their backpacks during the winter leaving the number of geolocators available for confirmation of migration at 15.

**Table 1:** Numbers of migrants and residents in each age and sex category.

	Migrants		Residents	
	Females	Males	Females	Males
Juvenile	11	5	14	30
Adult	5	13	4	34
Unknown age	4	0	11	0

**Figure 1:**
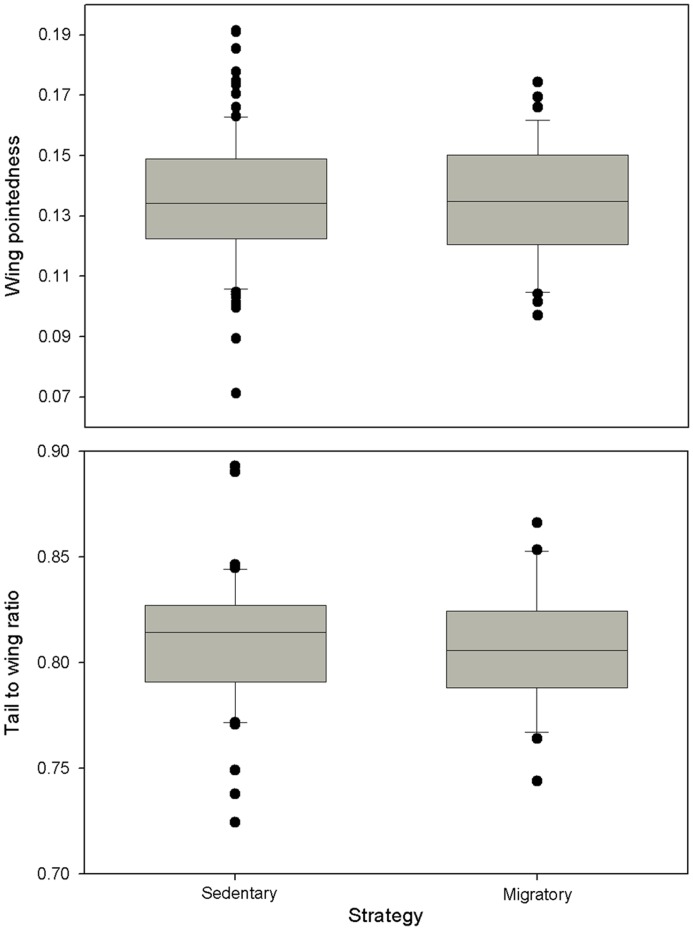
Comparison of the flight apparatus of migrant and sedentary blackbirds. Migrant and sedentary birds did not differ in either wing pointedness (A) or tail to wing ratio (B). Boxplots show the 5^th^ and 95^th^ percentiles. Circles indicate observations beyond the 5^th^ and 95^th^ percentiles.

Neither age nor the interactions between age and strategy or sex were significant in the initial models (wing pointedness: N = 116, age F = 0.002, P = 0.97; age * sex F = 2.580, P = 0.11; age * strategy F = 0.175, P = 0.68; age * sex * strategy F = 0.015, P = 0.90), (tail-wing ratio: N = 70, age F = 0.359, P = 0.55; age * sex F = 0.100, P = 0.75; age * strategy F = 1.657, P = 0.20; age * sex * strategy F = 0.238, P = 0.63). In the final models, with age removed, both wing pointedness and tail-wing ratio did not differ between migrants and residents, between the sexes, and there were no significant interactions between strategy and sex (wing pointedness: N = 131, strategy F = 0.028, P = 0.87; sex F = 0.247, P = 0.62; strategy * sex F = 0.651, P = 0.42) (Figure 1a), (tail-wing ratio: N = 75, strategy F = 0.261, P = 0.61; sex F = 0.040, P = 0.84; strategy * sex F = 0.369, P = 0.54 (Figure 1b). There were also no differences for either measure when we compared residents with only migrants that returned to the breeding grounds (wing pointedness: N = 113, strategy F = 0.076, P = 0.78; sex F = 0.000, P = 0.99; strategy * sex F = 1.001, P = 0.32), (tail-wing ratio: N = 68, strategy F = 0.337, P = 0.56; sex F = 0.221, P = 0.64; strategy * sex F = 0.064, P = 0.80). We found no relationship between individual wing pointendess scores and tail-wing ratio (N = 75, r_s_ = .037, P = 0.37).

## Discussion

In the current study, we found no differences in two primary indices of the external flight apparatus of coexisting migrant and resident European blackbirds (*Turdus merula*). Migrants and residents, independent of age and sex, had similar wing pointedness and tail-wing ratio scores, two indices that have commonly been found to correlate with migratory distance within songbird species [2]–[3], [9], [14], [20].

Compared with other migratory songbird species, blackbirds have relatively rounded wings and long tails. This is most likely an adaptation to aid maneuverability through their preferred forest habitat. Increased lifting surfaces (wings and tail) could aid blackbirds and other ‘ground foraging’ bird species to escape predators faster when foraging on the ground. However, based on previous studies [2]–[3], [9], [14], [20] we expected to find differences in the flight apparatus of migrants and residents. Migration is often thought to be an energetically expensive behavior that can be less costly with adaptations of wing and tail morphology [4], [19]. Cross-breeding experiments of migrant and resident songbirds from different populations resulted in mid-parent values of wing measures for offspring indicating high heritability of wing morphology [3], [20]. The current study is the first comparison of the flight apparatus of migrant and resident individuals from the same free living partially migratory population. Previous studies, which have compared populations of the same species that migrate different distances, have found correlations between migratory distance and wing pointedness and tail-wing ratio. Rolshausen et al. [14] compared sympatrically breeding blackcaps breeding at a migratory divide and found that a ∼550 km difference in migration distance correlated with a significant difference in flight apparatus measures. The two groups that were compared in Rolshausen et al. [14] spent the non breeding season in different habitats [13], which might provide additional pressure for divergence in their flight apparatus.

Our results indicate that either: 1) increases in the efficiency of the external flight apparatus for migration do not provide a strong enough benefit to be maintained among migrants in this population or 2) enough genetic recombination occurs between migrants and residents within the population to eliminate morphological differences. If sedentary individuals in the population initiate breeding prior to the arrival of migrants in the spring, then a sex bias in the migratory fraction of the population could act to reduce the effects of assortative mating. If there are less sedentary individuals of one sex, in this case females, then a fraction of the sedentary males would have to wait for migratory females to arrive for mating opportunities, resulting in genetic recombination between migrants and non migrants. Alternatively, non genetic factors could contribute to individual strategies. Although the combination of the results of captive studies and recaptures of migratory blackbirds up to 2000 km away from local breeding grounds during the winter strongly suggests a genetic contribution to migration strategies in blackbirds, early developmental effects and, potentially, differences in the individual sensitivities to environmental conditions might also result in differences in strategies [18]. If non genetic factors contribute to the migratory strategies of individuals, then selection could favor increased maneuverability through breeding habitat over increased efficiency in migration.

## Materials and Methods

### Ethics Statement

The study was approved by the Baden-Württemberg, Regierungspräsidium Freiburg Abteilung Umwelt (Aktenzeichen 55-8853. 17/0). Birds were removed from nets and processed as quickly as possible to minimize handling stress.

### Capture and Measurements

European blackbirds (*Turdus merula*) were captured over four years (2009–2012) in a mixed coniferous/deciduous forest in southern Germany (N 47° 47′, E 9° 2′). Birds were captured in spring and summer using 5–12 mist nets (12 m wide×3 m tall) that were opened between civil twilight and 12∶00 when weather permitted. Nets were placed on the edge of their breeding habitat next to known foraging areas and were checked every 30 min. After capture, age and sex of individuals were determined based on plumage differences [21]. Birds were classified in one of two age groups based on the age of their primary feathers (juvenile, adult). In the first calendar year, blackbirds do not molt either their primary or tail feathers. Juveniles were individuals in the first set of primaries when measured. Adults were all birds beyond the first set of primaries. Prior to their first pre-basic molt, the sex of hatch year blackbirds cannot be determined based on plumage differences. Fifty µl of blood was collected from hatch year birds from the brachial vein by venipuncture for molecular sex determination. We measured the length of each primary to the nearest 0.5 mm using a ruler specially designed with a fixed pin at the base. To measure a primary, the pin was pushed to the base of the feather, flush with the wing. Wing length (wing chord) was measured on the closed unflattened wing to the nearest 0.5 mm. The length of the tail was measured using the same method used for measuring primaries. The pin of the ruler was pushed against the base of the tail and aligned to the center rectrix.

### Backpack Attachment and Radiotracking

Mk 10S, Mk 12S and Mk20s geolocators (≤1.2 g; British Antarctic Survey, Cambridge, UK) connected to radio transmitters (≤2.6 g; Sparrow Systems, Fisher, IL, USA) with heat shrink tubing (≤0.4 g), were attached to birds via leg-loop harnesses. A range of harness sizes was built from 1 mm elastic beading cord to fit the naturally occurring body sizes of blackbirds in the population [22]. Each backpack weighed <5% of the mass of the individual that it was deployed on. Once a harness was fitted to a bird, it was inspected for appropriateness of fit. All birds were observed for as long as possible after release and throughout deployment to ensure normal behavior. All transmitters and geolocators were manufactured to last at least one year.

In order to identify individuals present at the breeding site and their departure dates, all birds (whenever present) were tracked using radio telemetry throughout the year. In the first year all birds were located twice per week from the date of capture until 1 December 2009. Beginning 1 December, birds were tracked once per week from the ground until recapture the following spring. In the second and third years, birds were tracked from the ground twice per week after capture until recapture the next spring, except from 20 December–10 January when birds were monitored exclusively from automated receivers. Ground tracking was done using the combination of either a handheld three element Yagi antenna (AF Antronics, Inc., Urbana, IL, USA) and AR 8200 MKIII handheld receiver (AOR U.S.A., Inc., Torrance, CA, USA) or a handheld H antenna (Andreas Wagener Telemetry Systems, Köln, DE) and a Yaesu VR 500 handheld receiver (Vertex Standard USA, Cypress, CA, USA). If an individual could not be located by ground tracking, aerial searches encompassing a 20 km radius (minimum) of the study site were performed using a Cessna airplane equipped with two H-antennas, one per wing, and two Biotrack receivers, one per antenna (Lotek, Newmarket, ON, Can). Individuals were classified as migrants after at least two searches from the air without a signal.

Three to five stationary automated receivers (Sparrow Systems, Fisher, IL, USA) were present at the study site throughout the study to monitor the presence of individuals, and departure and arrival dates [23]–[24]. Each automated receiver (ARU) searched for 16 frequencies every 60 seconds. Automated receivers were connected to H antennas (ATS, Isanti, MN, USA), mounted 3–6 m high. Not all birds were captured within range of an automated receiver; therefore, manual tracking was the only means for monitoring their presence. After a departure was identified, extensive ground and air tracking was done to confirm absences. After recapture, geolocators were used to confirm departure and arrival dates. To confirm departure dates, we identified the first date that longitude estimates diverged greater than 84 km from the breeding grounds and compared that date to the departure date identified by telemetry. We used 84 km because it is the known error in longitude estimates for geolocators on European blackbirds in the winter (see [25] for details of geolocator analysis). If geolocator data were not available and if a departure was not recorded on an ARU, the departure date was identified as the average date of the last observed date and first date missing. To identify return dates, ARU data were scanned to identify first observation. If a bird was first observed using manual telemetry, the arrival date was identified as the average date of the last date not observed during ground tracking and first date observed. To confirm arrival dates with geolocators, we identified the first date that longitude estimates were within 84 km of the breeding grounds in the spring and compared that date to the arrival date identified by telemetry. Occasionally, blackbirds in the study site that remained through the autumn moved from the study site during periods of extreme weather during the midst of winter (Fudickar and Partecke, unpublished data). For the purposes of this study, we were interested in differences between “autumn migrants” and “autumn residents” (individuals who stayed at the breeding site through the autumn and into winter). Re-sightings of autumn migrants in the following spring were typical for blackbirds breeding in rural habitat [26]. Therefore, all birds that departed during the autumn migratory period, regardless of whether they returned the following spring, were included in the analysis. Fifteen transmitter/geolocator backpacks were recovered from sedentary birds during the winter. Ten recoveries were identified as deaths because of either a recovered body or feathers. All fifteen sedentary birds from which we recovered backpacks were included in the analysis because they remained at the breeding grounds through the autumn migratory period.

### Statistical Analyses

Two indices of the flight apparatus were calculated to compare adaptations for energy-efficient flight of migrants and residents. We calculated an index of wing pointedness following Kipp [27]: wing pointedness = difference between longest primary and innermost primary/wing length. The second index, tail-wing ratio, was calculated by dividing tail length by wing length. Initially, we ran two separate general linear models (GLM) to test for differences in flight morphology of migrants and residents. To control for potential ontogenetic or sex differences in morphology, we included age and sex in the analyses. The first model tested for the effect of strategy (migrant or resident), sex, age, and all interactions on wing pointedness. The second model tested for the effect of strategy (migrant or resident), sex, age, and all interactions on tail-wing ratio. Because of the similarity in the coloration of primaries of juvenile and adult female blackbirds, we were unable to classify 15 females confidently into an age category. The initial models were limited to individuals for which age was determined. In order to test the effect of strategy and sex and their interaction on wing pointedness and tail-wing ratio for all individuals measured, we ran both models a second time, excluding age as a factor. To provide a conservative test of differences between migrants and residents we also ran the final models including only migrants that returned to the breeding grounds the following spring. We calculated a Spearman’s rank correlation coefficient to test for a correlation between wing pointedness and tail-wing ratio. All statistical analyses were performed in SPSS 15.0.
